# A theoretical model for predicting the startup performance of pumps as turbines

**DOI:** 10.1038/s41598-024-57693-9

**Published:** 2024-03-23

**Authors:** Yu-Liang Zhang, Yan-Juan Zhao, Zu-Chao Zhu

**Affiliations:** 1 College of Mechanical Engineering, Quzhou University, Quzhou, 324000 China; 2 College of Information Engineering, Quzhou College of Technology, Quzhou, 324000 China; 3 The Zhejiang Provincial Key Lab of Fluid Transmission Technology, Zhejiang Sci-Tech University, Hangzhou, 310018 China

**Keywords:** Energy science and technology, Engineering

## Abstract

Using the unsteady Bernoulli equation for the piping system and the angular momentum equation for the rotor, derives here a theoretical model to predict the startup performance of a pump as turbine (PAT). This model is effective for predicting the instantaneous evolution characteristics of the main performance parameters of PAT during startup, and these changings are initially faster and then slowly as a whole. The effect of the rotor moment of inertia and the final stabilized rotational speed of PAT on evolution characteristics of parameters is opposite. The rotational speed, head, hydraulic power, and conversion efficiency show a upward rising trend with the startup time, whereas the flow rate and hydraulic head loss display a downward trend.

## Introduction

The working principle of a centrifugal pump operating in reverse as a turbine is similar to that of a hydro-turbine, but the advantages of a pump as turbine (PAT) over hydro-turbine include wider ranges of flow rate and pressure head, a simpler structure, more-convenient operation, easier maintenance, and lower cost. Currently, PATs are used extensively to recover and utilize liquid residual pressure energy in processing industries and water distribution networks^1^.

As with a pump, a PAT has normal performance and transient performance: normal performance is the time-independent hydraulic performance when the PAT runs at constant rotational speed, while transient performance is the time-dependent hydraulic performance when the PAT operates at rapidly varying speed in the startup or shutdown periods. The transient performance is related to water hammer, which can cause a huge pressure to develop in the attached piping network, and this can damage the network or even the PAT itself.

To date, studies on PATs have been focused mainly on their normal hydraulic performance. For example, Wang et al. found that compared to a PAT with backward-curving blades, one with forward-curving blades has not only a flatter efficiency curve but also significantly higher efficiency^2^. Ashish et al. showed that a blunt shroud hub and blade trailing edges were effective for reducing the hydraulic losses of a PAT and improving its hydraulic efficiency, and modifying the angle of the backward-curving blades to reduce the hydraulic losses by 5–10%^3,4^. In an experimental study on an axial-flow PAT, Singh et al. observed a sharp drop in efficiency with more blades^5^; furthermore, Singh et al. showed that expanding the impeller outlet and draft tube could improve the efficiency of the PAT, and altering the blade profile also offered to improve hydraulic efficiency^6^. Yang et al. studied how the clearance size between the impeller and the volute affected the performance and pressure pulsation of a PAT; as the clearance increased, the efficiency of the PAT increased gradually beyond the best efficiency point, and the high-frequency pressure pulsation amplitude inside the volute decreased but the low-frequency pressure pulsation amplitude inside the impeller remained almost unchanged^7^. Li et al. simulated the flow inside a PAT and predicted its normal performance under high-viscosity flow conditions; the hydraulic efficiency decreased with increasing viscosity, and the output shaft power was determined mainly by the liquid density^8,9^. Furthermore, Li et al. noted that the influence of liquid viscosity on the performance of a PAT is more severe than that on a pump; the NPSHr (net positive suction head required) of a PAT increases with increasing liquid viscosity, but with a smaller increment.

To date, there have been relatively few investigations of transient PAT performance, such as the startup process. In Ref. 10, a generalized Euler equation for PATs was derived, and the transient performance of a PAT was measured when it was running in an atypical startup process, i.e., the rotational speed varied linearly with time as controlled by a frequency converter. Dimensionless analysis was conducted and the pressure head was calculated theoretically, showing that the transient characteristics of the PAT during this atypical startup process were due primarily to the increasing rotational speed rather than the flow inertia^10^.

As yet, there are no quick, robust, and accurate 1D methods for predicting transient PAT performance. Herein, a predictive model for PAT performance during startup is derived based on the unsteady Bernoulli equation for the piping system and the rotor angular momentum equation for the PAT. Using this model, the time-dependent hydraulic performance of the PAT is predicted effectively during the startup process and then analyzed. This study offers help to engineers for predicting the transient performance of PATs and assessing their hydraulic design.

## Theoretical model

This section begins by reviewing the 1D unsteady Bernoulli equation for a piping system for predicting the transient performance of a centrifugal pump, then this approach is applied to predict the transient performance of a PAT. The Bernoulli equation for the piping system is then combined with the rotor angular momentum equation to establish a theoretical model for simulating the transient performance during the PAT startup process.

### Unsteady Bernoulli equation for pump

Recently, studies on the normal hydraulic performance of centrifugal pumps have advanced rapidly^11,12^, and there has also been significant progress in investigating their transient performance during startup. Saito derived an expression of the pressure head during the transient startup process of a centrifugal pump by using the 1D unsteady Bernoulli equation for the piping system and the unsteady Euler head equation for the impeller in the pump, and the results showed that the proposed theoretical model was able to predict the pump head accurately^13^. Gao et al. established a mathematical model of the time-dependent hydraulic performance of a nuclear-reactor coolant pump during start-up period^14^. Thanapandi et al. suggested that the quasi-steady method could better predict the transient performance of centrifugal pumps during slow startup and shutdown^15^. Chalghoum et al. used the characteristic-curve method to approximately predict the pump flow–head characteristic curve at rated speed, finding that the starting time, valve opening, impeller diameter, and blade height had a significant impact on the pressure evolution process and performance curve^16^. Elaoud et al. conducted a study using the characteristic-curve method to investigate the transient characteristics of a pump, including its torque, flow rate, and changes in pressure within the upstream and downstream pipes under four different startup times^17^. Grover et al. divided the input torque into four parts, i.e., pipeline friction loss, pump internal loss, rotor acceleration, and pipeline fluid acceleration, and using the torque balance equation, they calculated numerically how the rotor rotational inertia and pipeline fluid inertia influence the transient characteristics during startup^18^. Farhadi et al. introduced the concept of the effective energy ratio (the ratio of the kinetic energy of the cooling fluid to that of the rotating components inside the pump) and established a mathematical model for predicting the transient performance during startup of a reactor pump^19^. Those studies used the following 1D unsteady Bernoulli equation for the piping system between the pump inlet and outlet^20^:

1 $$ \left( \frac{L}{A} \right)_{{{\text{cl}}}} \frac{{\text{d}q}}{{\text{d}t}} + k_{{\text{cl}}} \frac{{q^{2} }}{2\rho } + \text{g}(\rho \Delta z)_{{{\text{cl}}}} = \rho \text{g}h_{\text{p}} $$

where *L* and *A* are the length and cross-sectional area, respectively, of a segment of the flow passage in the piping loop, the subscript “cl” indicates the whole loop system, including the valves, tank, bends, etc., (*L*/*A*)_cl_ is the geometrical inertia, *q* is the mass flow rate, g is the gravitational acceleration, and *k*_cl_ is the resistance coefficient. The second item on the left-hand side represents the friction pressure drop, local pressure drop, and fluid acceleration drop, where ∆*z* is the potential difference, *ρ* is the average density, and *h*_p_ is the pump head.

**Figure 1:**
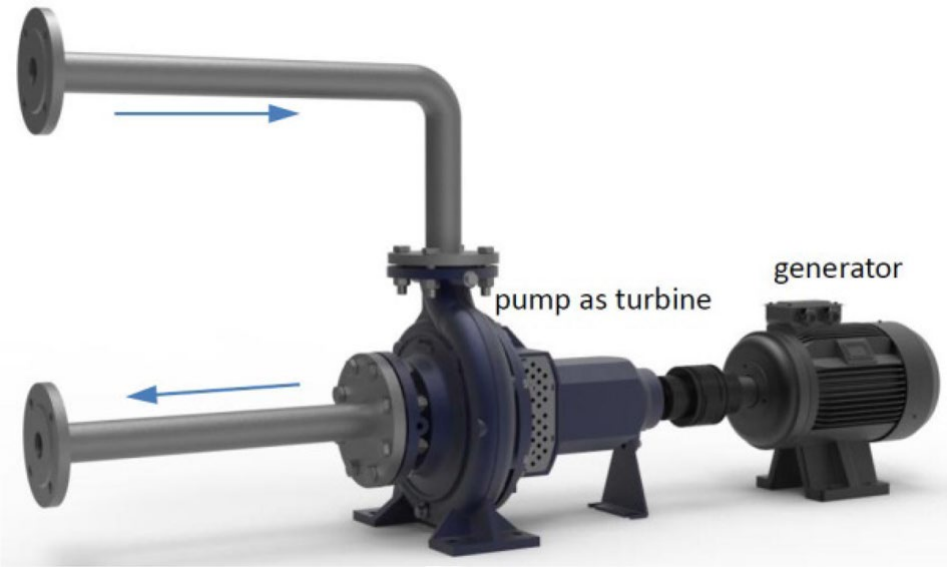
Working diagram of pump as turbine (PAT).

Working machines such as pumps, fans, and compressors do mechanical work on the fluid therein, which results in an increase in fluid energy, thus *h*_p_ on the right-hand side of Eq. (1) is positive. For prime movers such as PATs (Fig. 1), hydro-turbines, and gas turbines, the fluid does work on the machine, which makes the fluid energy decrease, so *h*_p_ on the right-hand side of Eq. (1) is negative.

### Unsteady Bernoulli equation for PAT

Consider a PAT driven by a constant-pressure source* P*_0_. In a piping system including the constant-pressure source, valves, pipes, and bends, Eq. (1) becomes

2 $$ \left( \frac{L}{A} \right)_{{{\text{cl}}}} \frac{{\text{d}q}}{{\text{d}t}} + k_{{\text{cl}}} \frac{{q^{2} }}{2\rho } + \text{g}(\rho \Delta z) + \rho \text{g}h_{{\text{t}}} { = }P_{0} $$

where *P*_0_ is the total pressure at the pressure source, which represents the total energy of the incoming flow, and *h*_t_ is the head between the PAT inlet and outlet, here *h*_t_ replaces − *h*_p_. Neglecting the effect of gravitational potential energy, Eq. (2) simplifies to

3 $$ \left( \frac{L}{A} \right)_{{{\text{cl}}}} \frac{{\text{d}q}}{{\text{d}t}} + k_{{\text{cl}}} \frac{{q^{2} }}{2\rho } + \rho \text{g}h_{{\text{t}}} { = }P_{0} $$

and the first term on the left-hand side can be neglected in the case of the steady working conditions of the PAT. As is known, under steady working conditions, the mass flow rate (*q*_0_) and the head (*h*_t0_) of a PAT can be determined in advance experimentally, i.e.,

4 $$ k_{{\text{cl}}} \frac{{q_{0}^{2} }}{2\rho } + \rho \text{g}h_{{{\text{t}}0}} { = }P_{0} $$

Therefore, the flow resistance coefficient of the piping system is determined by

5 $$ k_{{\text{cl}}} { = }\frac{{2\rho P_{0} }}{{q_{0}^{2} }} - \frac{{{2}\rho^{2} \text{g}h_{{{\text{t}}0}} }}{{q_{0}^{2} }} $$

and substituting Eqs. (5) into(3) gives

6 $$ h_{{\text{t}}} = \frac{{P_{0} }}{{\rho \text{g}}} - \frac{{\left( {\tfrac{L}{A}} \right)_{{\text{cl}}} }}{{\rho \text{g}}}\frac{{\text{d}q}}{{\text{d}t}} - \left( {\frac{{P_{0} }}{{\rho \text{g}}} - h_{{\text{t0}}} } \right)\frac{{q^{2} }}{{q_{0}^{2} }} $$

which can be rewritten according to liquid column heights as

7 $$ h_{{\text{t}}} = H_{0} - h_{\text{a}} - h_{\text{f}} $$

where *H*_0_ is the total head representing the total energy of the incoming flow, *h*_a_ is the fluid acceleration head (FAH), and *h*_f_ is the head consumed to overcome frictional resistance and minor hydraulic losses across the bends and valves. Comparing Eqs. (6) and (7), we have

8 $$ h_{a} = \frac{{\left( {\tfrac{L}{A}} \right)_{{\text{cl}}} }}{{\rho \text{g}}}\frac{{\text{d}q}}{{\text{d}t}} $$

9 $$ h_{\text{f}} = \left( {\frac{{P_{0} }}{{\rho \text{g}}} - h_{{\text{t0}}} } \right)\frac{{q^{2} }}{{q_{0}^{2} }} $$

Next, we consider the angular momentum equation for the PAT rotor during startup without any control. It is assumed that the torque applied on the rotor is proportional to the square of the rotational speed of the PAT rotor, and the angular momentum equation is^14^

10 $$ I\frac{{\text{d}\omega }}{{\text{d}t}} = C\omega_{0}^{2} - C\omega^{2} $$

where *I* is the moment of inertia of the PAT rotor, *ω* is the angular speed of the rotor, *ω*_0_ is the angular speed under normal/steady operating conditions, and *C* is a constant of proportionality. The initial moment of startup corresponds to *t* = 0 and *ω* = 0. The analytical solution of Eq. (10) is written as^14^

11 $$ \omega \left( t \right) = \omega_{0} \tanh \left( {\frac{{C\omega_{0} }}{I}t} \right) $$

Next, we combine Eqs. (6) and (11) to generate a 1D ordinary differential equation for the transient performance during startup. Dividing both sides of Eq. (6) by *h*_t0_ gives

12 $$ \frac{{h_{{\text{t}}} }}{{h_{{\text{t}0}} }} = \frac{{P_{0} }}{{\rho \text{g}h_{{\text{t}0}} }} - \frac{{\left( {\tfrac{L}{A}} \right)_{{\text{cl}}} }}{{\rho \text{g}h_{{\text{t}0}} }}\frac{{\text{d}q}}{{\text{d}t}} - \left( {\frac{{P_{0} }}{{\rho \text{g}h_{{\text{t}0}} }} - 1} \right)\frac{{q^{2} }}{{q_{0}^{2} }} $$

and assuming that the head across the PAT is proportional to the square of its rotational speed^21,22^, Eq. (12) becomes

13 $$ \frac{{\left( {\tfrac{L}{A}} \right)_{{\text{cl}}} }}{{\rho \text{g}h_{{\text{t}0}} }}\frac{{\text{d}q}}{{\text{d}t}}{ + }\left( {\frac{{P_{0} }}{{\rho \text{g}h_{{\text{t}0}} }} - 1} \right)\frac{{q^{2} }}{{q_{0}^{2} }} - \frac{{P_{0} }}{{\rho \text{g}h_{{\text{t}0}} }}{ = } - \left( {\frac{\omega }{{\omega_{0} }}} \right)^{2} $$

Substituting Eq. (11) into Eq. (13) gives

14 $$ \frac{{\left( {\tfrac{L}{A}} \right)_{{\text{cl}}} }}{{\rho \text{g}h_{{\text{t}0}} }}\frac{{\text{d}q}}{{\text{d}t}}{ + }\left( {\frac{{P_{0} }}{{\rho \text{g}h_{{\text{t}0}} }} - 1} \right)\frac{{q^{2} }}{{q_{0}^{2} }} - \frac{{P_{0} }}{{\rho \text{g}h_{{\text{t}0}} }}{ = } - \tanh^{2} \left( {\frac{{C\omega_{0} }}{I}t} \right) $$

Equations (6), (8), (9), (11), and (14) have five unknowns (*q*, *ω*, *h*_a_, *h*_f_, *h*_t_) and so can be solved numerically for a given time *t* in MATLAB. Not only can this mathematical model be used for predicting the time-dependent performance of a PAT during startup, it can also be applied to other transient processes such as shutdown and adjusting the rotational speed.

## Results

### Study cases

A piping system including a PAT is used to calculate and analyze the startup transient behavior by means of the method proposed above. The values of the various parameters are given in Table 1.

**Table 1:** Parameters used in predicting transient performance.

Component	Parameter	Value
Pipe	*L *(m)	20
	*A *(m^2^)	0.002
PAT	*n*_0_ (r/min)	1432
	*q*_0_ (m^3^/h)	36
	*h*_t0_ (m)	20
Rotor	*Ι* (kg m^2^)	1000
	*C*	2
Liquid	*ρ* (kg/m^3^)	1000
	*P*_0_ (MPa)	0.5
	*g* (m/s^2^)	9.8

Usually, the flow rate and pressure of the coming flow are approximately constant in practical applications. The selected PAT is installed in a piping system, which is also usually fixed. Therefore, the characteristic parameters of the selected PAT itself (e.g., the rotor moment of inertia *Ι*, steady rotational speed *n*_0_, and PAT head *h*_t0_) may have a significant effect on the startup process. To reveal how these parameters influence the transient performance, the values of *Ι*, *n*_0_, and *h*_t0_ are varied as given in Table 2.

**Table 2:** Variations of *Ι*, *n*_0_, and *h*_t0_.

Parameter	Value					
*Ι*	250	500	750	1000	1250	1500
*h*_t0_	5	10	15	20	25	30
*n*_0_	250	500	750	1000	1450	2950

### Rotational speed

**Figure 2:**
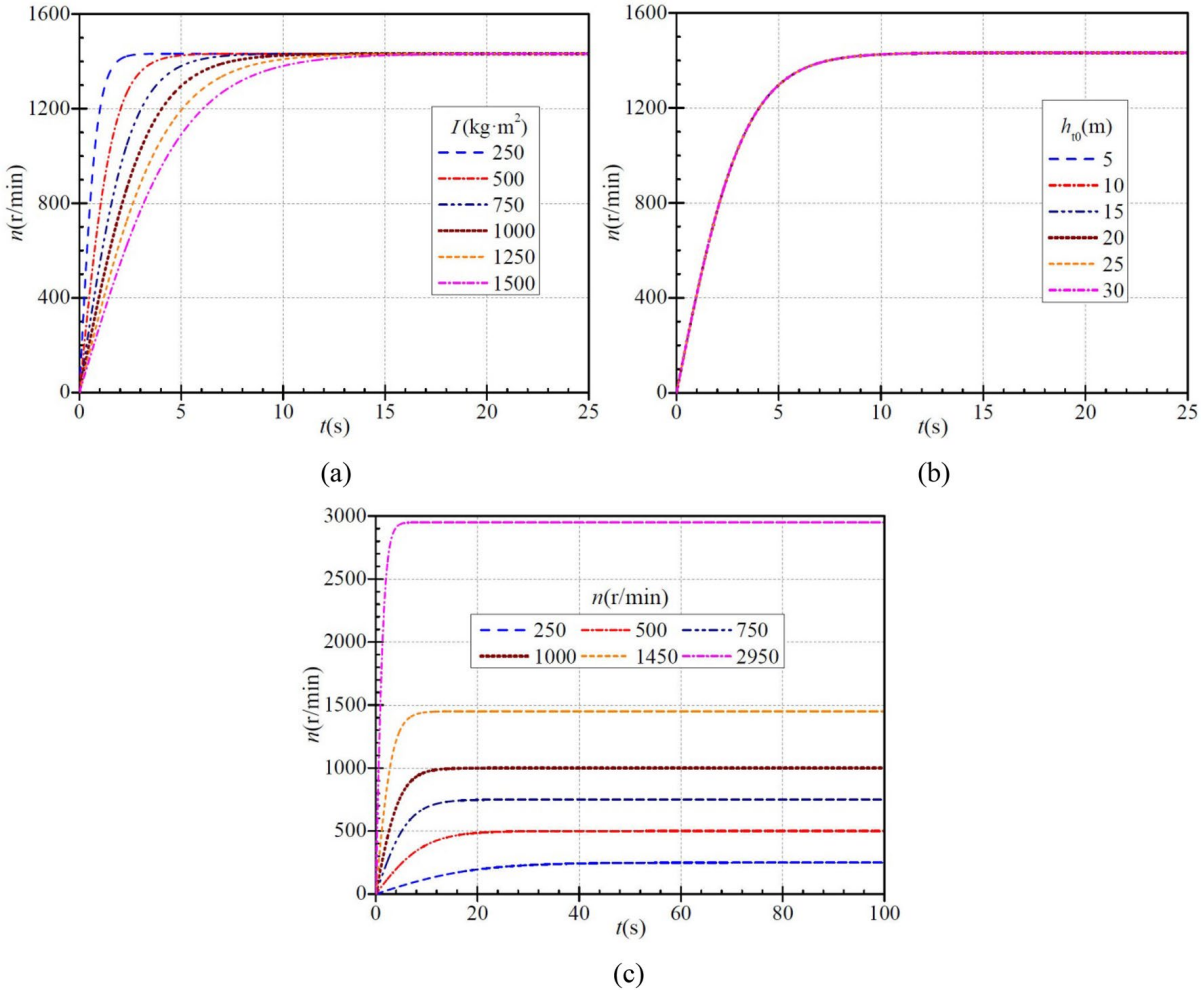
Instantaneous characteristics of rotational speed during PAT startup: (**a**) effect of moment of inertia; (**b**) effect of steady head; (**c**) effect of steady rotational speed.

Figure 2a shows that the time required for the rotational speed to reach a steady value is ca. 3.7 s, 7.4 s, 11.0 s, 14.7 s, 18.4 s, and 22.0 s when the moment of inertia of the PAT rotor is 250 kg∙m^2^, 500 kg∙m^2^, 750 kg∙m^2^, 1000 kg∙m^2^, 1250 kg∙m^2^, and 1500 kg∙m^2^, respectively. As the moment of inertia of the impeller increases, the rotational speed increases less rapidly, i.e., it takes longer to reach a stable value, which is perfectly consistent with the actual situation. The time required to reach the stable rotational speed is ca. 1.0 s, 2.0 s, 3.0 s, 4.0 s, 5.0 s, and 6.0 s, respectively, when the rotational speed rises to 1200 r/min in the six cases. At this time, the increase in rotational speed has been completed more than 80%, while the ratios of the required time to total required time are only 27%, 27%, 27.3%, 27.2%, 27.2%, and 27.3%, respectively. Obviously, during PAT startup, the rotational speed tends to increase faster and then slower with time, and the above influence of the moment of inertia on the PAT rotational speed is fully determined by Eq. (11).

Both Fig. 2b and Eq. (11) show clearly that the rise characteristic of rotational speed is independent of the stabilized PAT head. Actually, in Fig. 2b, five curves are displayed. As mentioned above, the rotational speed are not related to the stable PAT head. Therefore, five curves overlap. As a results, it looks like a curve. Figure 2c shows that as the stabilized PAT rotational speed increases, the rotational speed rise becomes more rapid, i.e., the time required for completing startup tends to decrease. When the stabilized rotational speeds are 250 r/min, 500 r/min, 750 r/min, 1000 r/min, 1450 r/min, and 2950 r/min, the time required for the rotational speed to rise to the stabilized value is ca. 59.3 s, 52.8 s, 35.2 s, 26.4 s, 21.1 s, and 7.1 s, respectively.

### Flow rate

**Figure 3:**
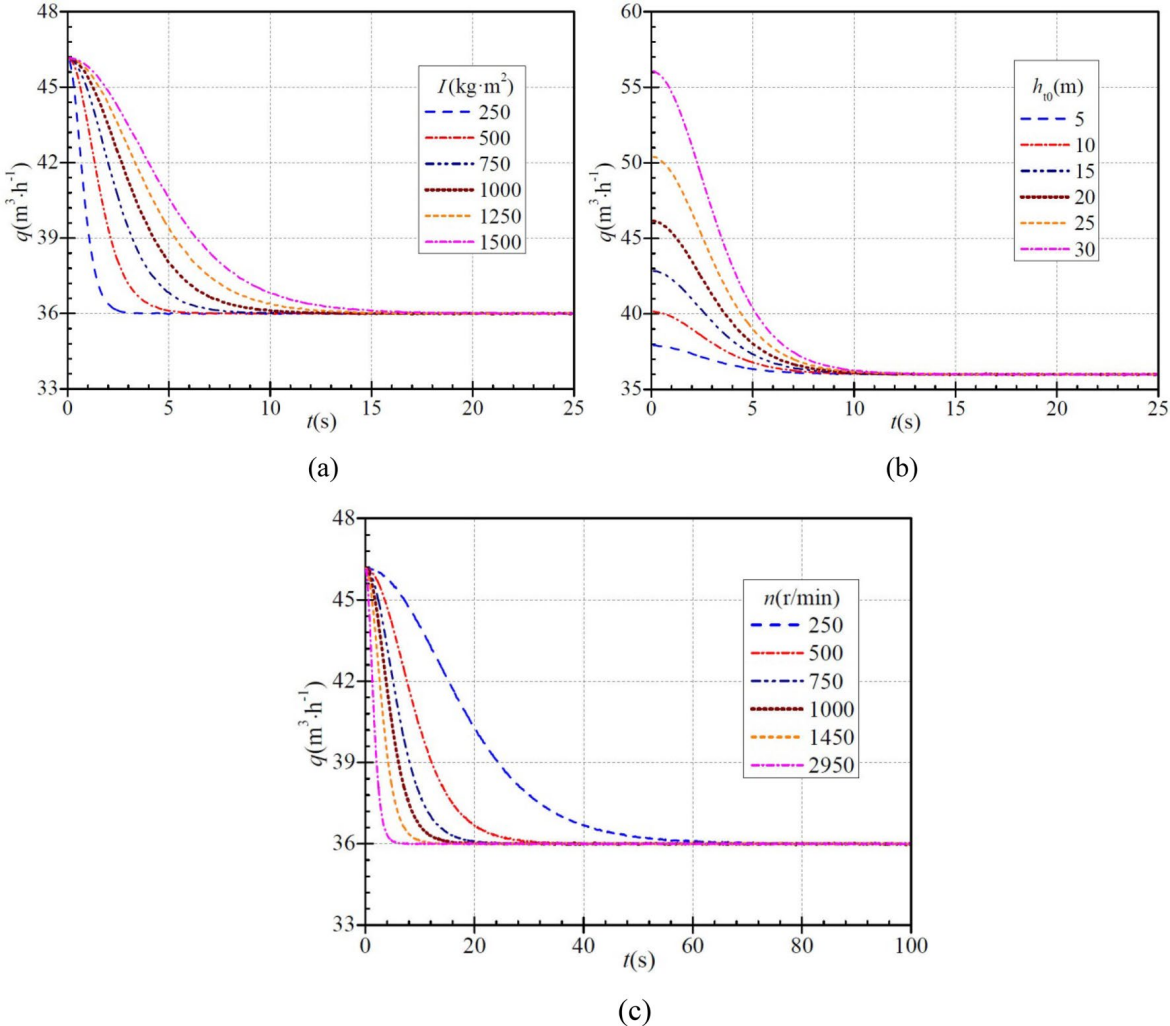
Instantaneous characteristics of flow rate during PAT startup: (**a**) effect of moment of inertia; (**b**) effect of steady head; (**c**) effect of steady rotational speed.

Figure 3a shows that for a moment of inertia of 250 kg∙m^2^, 500 kg∙m^2^, 750 kg∙m^2^, 1000 kg∙m^2^, 1250 kg∙m^2^, and 1500 kg∙m^2^, the initial flow rates through the PAT are always 46.2 m^3^/h in six cases, and then decreases with time to the final stabilized flow rate of 36.0 m^3^/h. The time required for this process is ca. 2.9 s, 6.7 s, 9.2 s, 13.5 s, 16.0 s, and 18.6 s, respectively, which shows that as the moment of inertia increases, the flow rate decreases less rapidly, and the time required to reach the stabilized value tends to increase. When the instantaneous flow rate decreases to 38.0 m^3^/h, 80% of the decrease in flow rate has been completed. The time required for this in the six cases is ca. 1.3 s, 2.5 s, 3.7 s, 5.0 s, 6.2 s, and 7.5 s, respectively, and the ratios to the total required time are ca. 41.4%, 37.3%, 40.2%, 37.0%, 38.9%, and 40.3%, respectively. As can be seen, the instantaneous flow rate decreases faster initially and then more slowly regardless of any a case.

Figure 3b shows how the stabilized head of the PAT affects the instantaneous flow rate. When the stabilized heads are 5 m, 10 m, 15 m, 20 m, 25 m, and 30 m, the initial flow rates through the PAT are ca. 37.9 m^3^/h, 40.2 m^3^/h, 42.8 m^3^/h, 46.2 m^3^/h, 50.4 m^3^/h, and 56.1 m^3^/h, respectively. As can be seen, the initial flow rate increases with increasing stabilized head of the PAT. The initial flow rate then starts to decrease gradually to the final stabilized value of 36.0 m^3^/h, and the time required for this is the six cases is ca. 9.2 s, 10.9 s, 11.7 s, 13.2 s, 14.6 s, and 15.2 s, respectively, which shows that with increasing stabilized head, the total time required for the flow rate to decrease tends to increase.

Figure 3c shows that when the final stabilized rotational speeds are 250 r/min, 500 r/min, 750 r/min, 1000 r/min, 1450 r/min, and 2950 r/min, the initial flow rate through the PAT is ca. 46.2 m^3^/h and then decreases gradually to the final steady flow rate of 36 m^3^/h. The time required to complete this process is ca. 71.2 s, 34.7 s, 24.6 s, 16.5 s, 12.2 s, and 6.4 s, respectively, which shows that with increasing stabilized rotational speed, the flow rate decreases more rapidly, and the time required to reach the stabilized value decreases. When the instantaneous flow rate decreases to 38 m^3^/h, the flow-rate decrease has also completed 80%. The time required for this in the six cases is ca. 28.9 s, 14.4 s, 9.6 s, 7.2 s, 4.9 s, and 2.4 s, and the ratios to the total required time are ca. 40.6%, 41.5%, 39.0%, 43.6%, 40.2%, and 37.5%, respectively. As can be seen, the instantaneous flow rate shows the same trend of decreasing faster initially and then more slowly in six cases.

### Head

**Figure 4:**
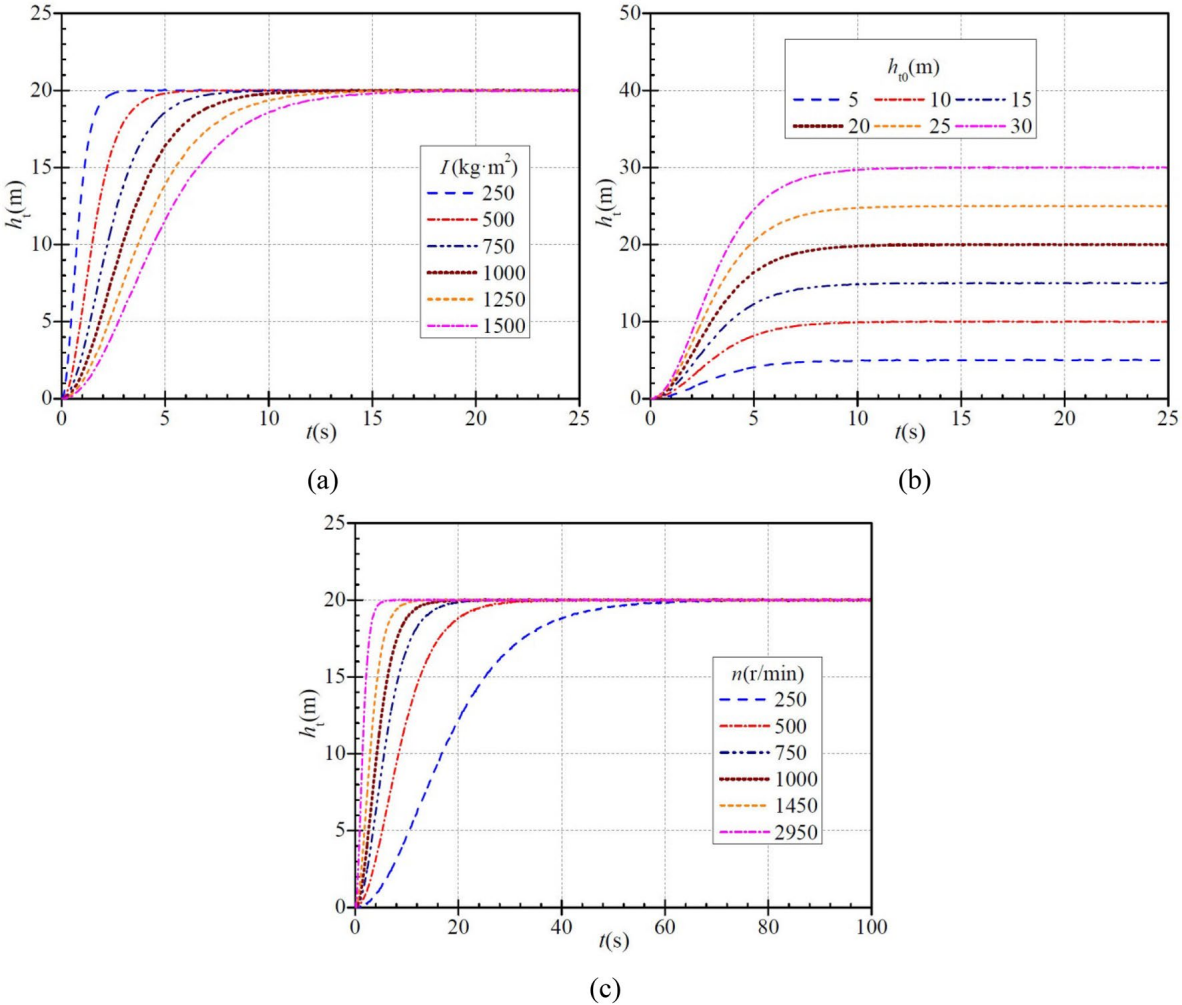
Instantaneous characteristics of head during PAT startup: (**a**) effect of moment of inertia; (**b**) effect of steady head; (**c**) effect of steady rotational speed.

Figure 4a shows that the instantaneous head tends to increase gradually with time for any moment of inertia. The time required for the instantaneous head to reach the stabilized value is ca. 3.9 s, 6.7 s, 10.5 s, 13.3 s, 14.7 s, and 19.8 s for a moment of inertia of 250 kg∙m^2^, 500 kg∙m^2^, 750 kg∙m^2^, 1000 kg∙m^2^, 1250 kg∙m^2^, and 1500 kg∙m^2^, respectively, showing that the instantaneous head reaches the stabilized value less rapidly with increasing moment of inertia. As can be seen, with increasing moment of inertia, the time required for the instantaneous head to reach the stabilized value tends to increase, i.e., the rise becomes slower. When the instantaneous head rises to 16 m, the head rise has completed 80%. At this time, the time required for the head rise in the six cases is ca. 1.2 s, 2.4 s, 3.6 s, 4.8 s, 6.0 s, and 7.2 s, and the ratios of the required time to the total time are ca. 30.8%, 35.8%, 34.3%, 36.1%, 40.8%, and 36.4%, respectively. As can be seen, the instantaneous head increases faster initially and then more slowly regardless of any a case.

Figure 4b shows how the stabilized head of the PAT affects the instantaneous head. For each stabilized head, the instantaneous head increases gradually with time. When the stabilized heads are 5 m, 10 m, 15 m, 20 m, 25 m, and 30 m, the time required for the instantaneous heads to rise to the stabilized values is ca. 10.2 s, 11.7 s, 11.9 s, 13.3 s, 14.0 s, and 13.8 s, respectively. When the stabilized head is small (< 25 m), the time required for the instantaneous head to rise tends to be slightly prolonged. When the instantaneous head rises to 2.5 m, 5 m, 7.5 m, 10 m, 12.5 m, and 15 m in the six cases, the head rise has completed 50%, and the time required for this is ca. 2.95 s, which corresponds to the ratios of this time to the total time of ca. 28.9%, 25.2%, 24.8%, 22.2%, 21.1%, and 21.4%, respectively. This indicates that the rise in instantaneous head is always faster and then slower in six cases.

Figure 4c shows that for each stabilized rotational speed, the instantaneous head increases gradually with time. When the stabilized rotational speeds are 250 r/min, 500 r/min, 750 r/min, 1000 r/min,1450 r/min, and 2950 r/min, the time required for the instantaneous head to rise to the stabilized value is ca. 73.6 s, 37.6 s, 25.3 s, 19.0 s, 12.2 s, and 7.2 s, respectively. With increasing stabilized rotational speed, the instantaneous head rises faster, and as can be seen, the rise of instantaneous head again shows the trend of being faster and then slower.

### Fluid acceleration head

**Figure 5:**
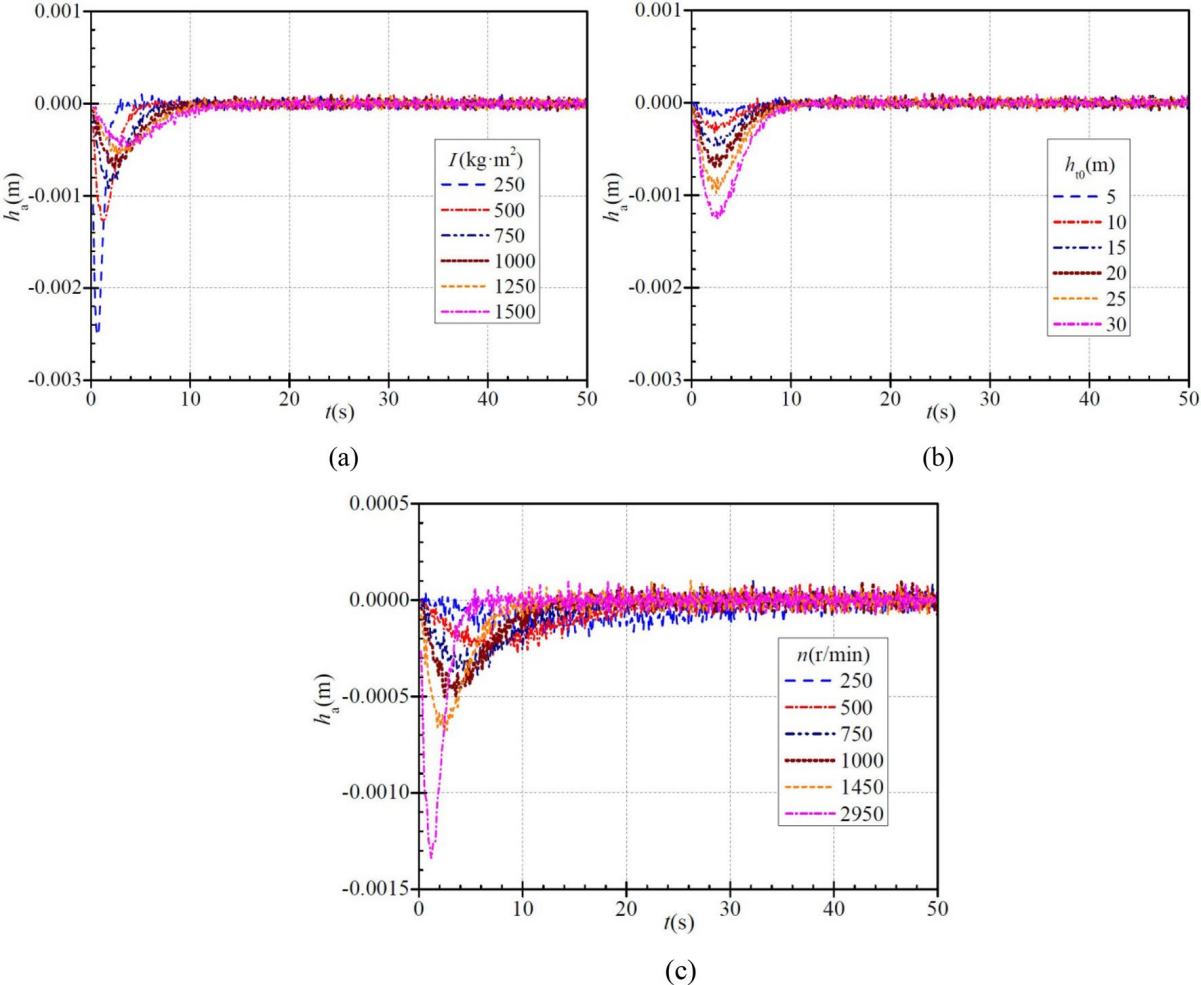
Instantaneous characteristics of fluid acceleration head (FAH) during PAT startup: (**a**) effect of moment of inertia; (**b**) effect of steady head; (**c**) effect of steady rotational speed.

Figure 5 shows how the three parameters affect the FAH during PAT startup. In each case, the calculated FAH is basically negative because during PAT startup, the flow rate through the PAT decreases gradually (Fig. 2), and so acceleration is actually deceleration and thus the FAH is negative. Also, in each case the absolute value of instantaneous FAH tends to increase and then decrease, and its maximum value is only 0.00255 m, which is negligibly small.

Figure 5a shows that the FAH rises to the maximum value of 0.00255 m, 0.00128 m, 0.00097 m, 0.00075 m, 0.00061 m, and 0.00056 m for a moment of inertia of 250 kg∙m^2^, 500 kg∙m^2^, 750 kg∙m^2^, 1000 kg∙m^2^, 1250 kg∙m^2^, and 1500 kg∙m^2^, respectively, and the time required is ca. 0.6 s, 1.2 s, 1.8 s, 2.4 s, 2.6 s, and 3.1 s, respectively. With increasing moment of inertia, the maximum absolute value of the FAH tends to decrease, while the time required tends to increase.

Figure 5b shows that when the stabilized head is 5 m, 10 m, 15 m, 20 m, 25 m, and 30 m, the maximum absolute instantaneous FAH is ca. 0.00021 m, 0.0004 m, 0.0005 m, 0.00075 m, 0.0011 m, and 0.0013 m, respectively, and the time taken is ca. 2.0 s, 2.3 s, 2.8 s, 2.4 s, 2.5 s, and 2.6 s, respectively. As can be seen, with increasing stabilized head, the maximum absolute value of the FAH shows an increasing trend, while the difference in the time required is not significant.

Figure 5c shows that when the stabilized rotational speeds are 250 r/min, 500 r/min, 750 r/min, 1000 r/min, 1450 r/min, and 2950 r/min, the FAH rises to the maximum value of ca. 0.00023 m, 0.00029 m, 0.00042 m, 0.00055 m, 0.00072 m, and 0.00136 m, respectively, and the time required is ca. 5.7 s, 4.8 s, 3.7 s, 2.5 s, 1.8 s, and 1.2 s, respectively. With increasing stabilized rotational speed, the maximum FAH increases and the time required decreases.

### Hydraulic head loss

**Figure 6:**
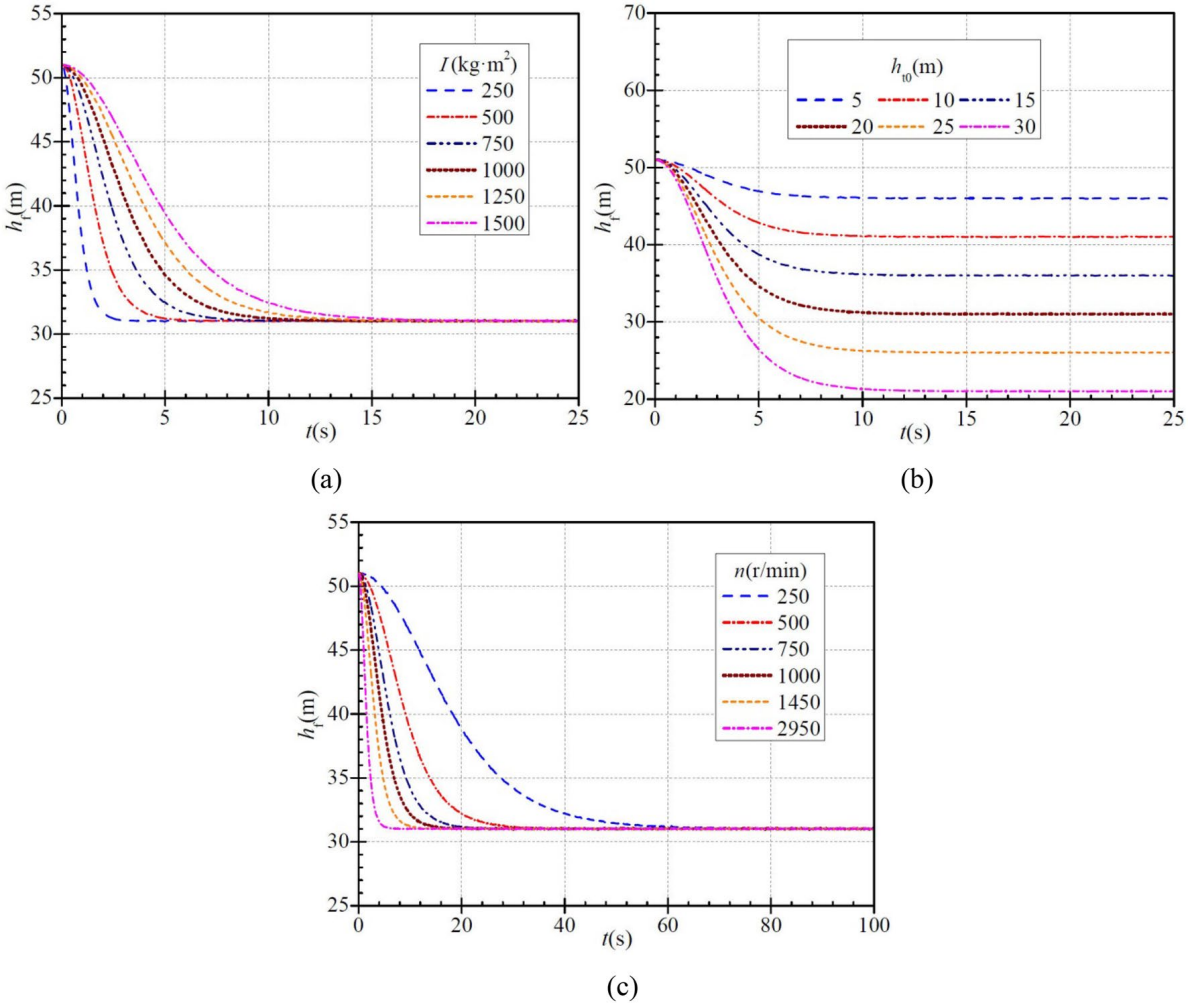
Instantaneous characteristics of hydraulic head loss (HHL) during PAT startup: (**a**) effect of moment of inertia; (**b**) effect of steady head; (**c**) effect of steady rotational speed.

Figure 6a shows that for moments of inertia of 250 kg∙m^2^, 500 kg∙m^2^, 750 kg∙m^2^, 1000 kg∙m^2^, 1250 kg∙m^2^, and 1500 kg∙m^2^, the initial instantaneous hydraulic head loss (HHL) during PAT startup is ca. 51.0 m and then decreases gradually to a final stable value of ca. 31.0 m. The time required to complete this process in the six cases is ca. 2.9 s, 5.9 s, 8.7 s, 11.4 s, 14.3 s, and 17.3 s, respectively. With increasing moment of inertia, the time required for the HHL to reach the stabilized value tends increase, i.e., the decrease becomes slower. When the HHL drops to 35 m, 80% of the decrease has been completed. The time required for this in the six cases is ca. 1.2 s, 2.4 s, 3.6 s, 4.8 s, 6.0 s, and 7.2 s, respectively, and the ratios of the required time to the total time required are ca. 41.4%, 40.7%, 41.4%, 42.1%, 42.0%, and 41.6%, respectively. It is found that the HHL also decreases faster initially and then more slowly regardless of any a case.

Figure 6b shows that when the stabilized PAT heads are 5 m, 10 m, 15 m, 20 m, 25 m, and 30 m, the initial instantaneous HHL is ca. 51.0 m and then decreases gradually to the stabilized value of ca. 46.0 m, 41.0 m, 36.0 m, 31.0 m, 26.0 m, and 21.0 m, respectively, and the time required for this is ca. 11.3 s, 11.6 s, 13.1 s, 14.3 s, 14.5 s, and 16.5 s, respectively. With increasing stabilized PAT head, the final stabilized HHL decreases while the time required increases. As can be seen, the HHL also decreases faster initially and then more slowly in six cases.

At different stabilized rotational speeds, the HHL decreases gradually with time, as shown in Fig. 6c. When the stabilized rotational speeds are 250 r/min, 500 r/min, 750 r/min, 1000 r/min, 1450 r/min, and 2950 r/min, the initial instantaneous HHL during startup is ca. 51.0 m and then decreases gradually to the stabilized value of ca. 31.0 m, and the time required for this is ca. 76.2 s, 37.6 s, 27.0 s, 19.0 s, 13.7 s, and 7.8 s, respectively. This indicates that with increasing stabilized rotational speed, the time required for the HHL to stabilize decreases, i.e., it falls more rapidly. Again, the HHL decreases faster initially and then more slowly.

### Hydraulic power

**Figure 7:**
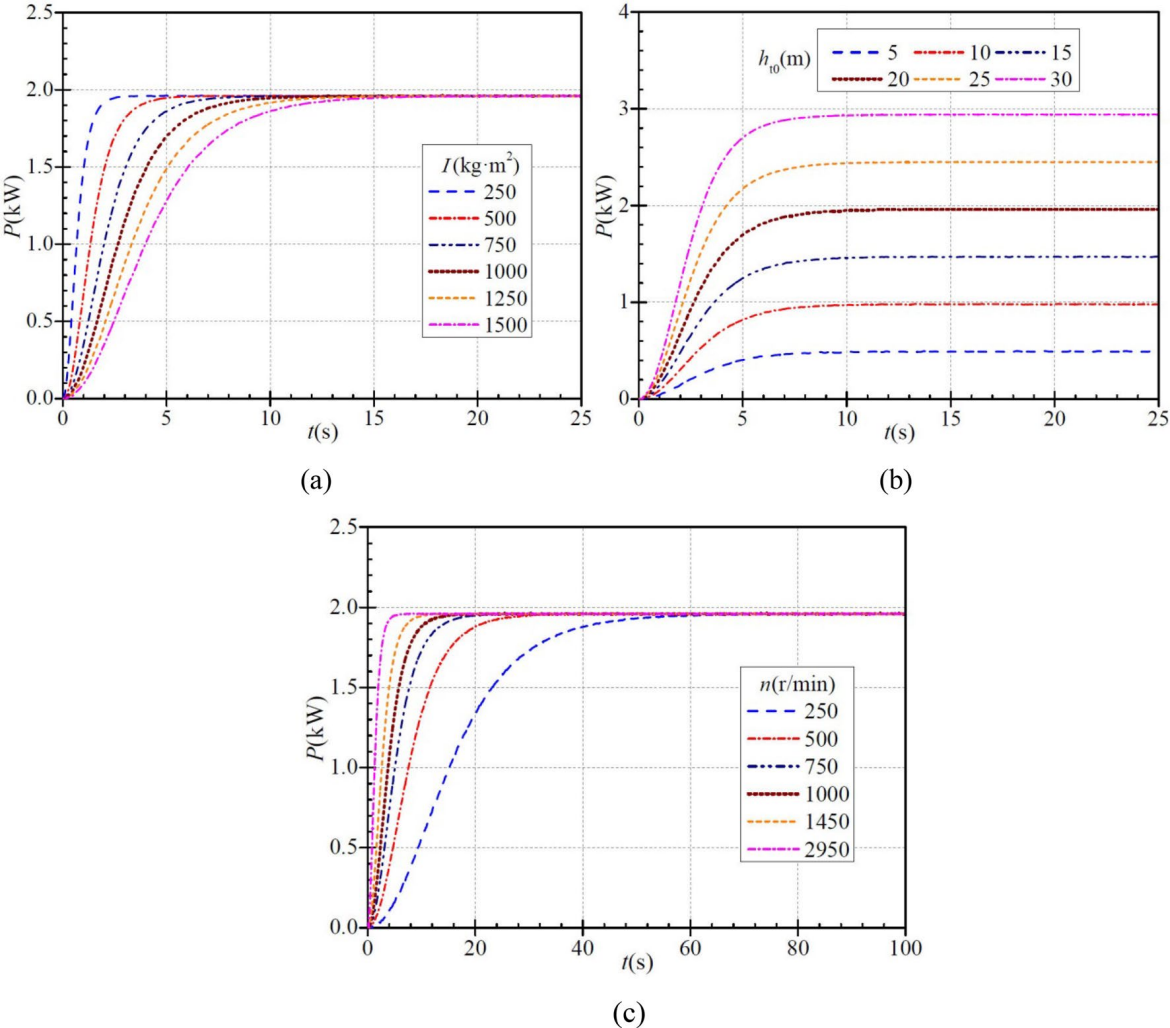
Instantaneous characteristics of hydraulic power during PAT startup: (**a**) effect of moment of inertia; (**b**) effect of steady head; (**c**) effect of steady rotational speed.

The hydraulic power (*P*) of the PAT is defined as

15 $$ P = \rho \text{g}h_{\text{t}} q $$

and Fig. 7 shows how the instantaneous hydraulic power evolves with time in the startup process. For each moment of inertia, the hydraulic power increases gradually with time, as shown in Fig. 7a. For moments of inertia of 250 kg∙m^2^, 500 kg∙m^2^, 750 kg∙m^2^, 1000 kg∙m^2^, 1250 kg∙m^2^, and 1500 kg∙m^2^, the hydraulic power rises to a steady value of ca. 1.960 kW in a time of 3.8 s, 6.6 s, 10.5 s, 13.2 s, 14.7 s, and 19.8 s, respectively. As the moment of inertia increases, so does the time required for the hydraulic power to reach a stabilized value, i.e., the rise becomes slower. Again, the hydraulic power during PAT startup increases faster initially and then more slowly in six cases.

Figure 7b shows how the PAT stabilized head affects the hydraulic power. Under each stabilized head, the hydraulic power increases gradually with time. When the stabilized heads are 5 m, 10 m, 15 m, 20 m, 25 m, and 30 m, the hydraulic power rises to the stabilized values of ca. 0.489 kW, 0.980 kW, 1.470 kW, 1.960 kW, 2.450 kW, and 2.940 kW, respectively, in a time of ca. 9.2 s, 10.9 s, 11.7 s, 11.4 s, 14.0 s, and 13.8 s, respectively. Again, the hydraulic power during PAT startup increases faster initially and then more slowly.

Figure 7c shows that the hydraulic power during PAT startup increases gradually with time for each stabilized rotational speed. When the stabilized rotational speeds are 250 r/min, 500 r/min, 750 r/min,1000 r/min, 1450 r/min, and 2950 r/min, the hydraulic power rises to the stabilized value of ca. 1.960 kW in a time of ca. 76.2 s, 36.6 s, 25.3 s, 19.0 s, 12.2 s, and 6.4 s, respectively. As can be seen, with increasing stabilized rotational speed, the hydraulic power increases faster initially and then more slowly.

### Conversion efficiency

**Figure 8:**
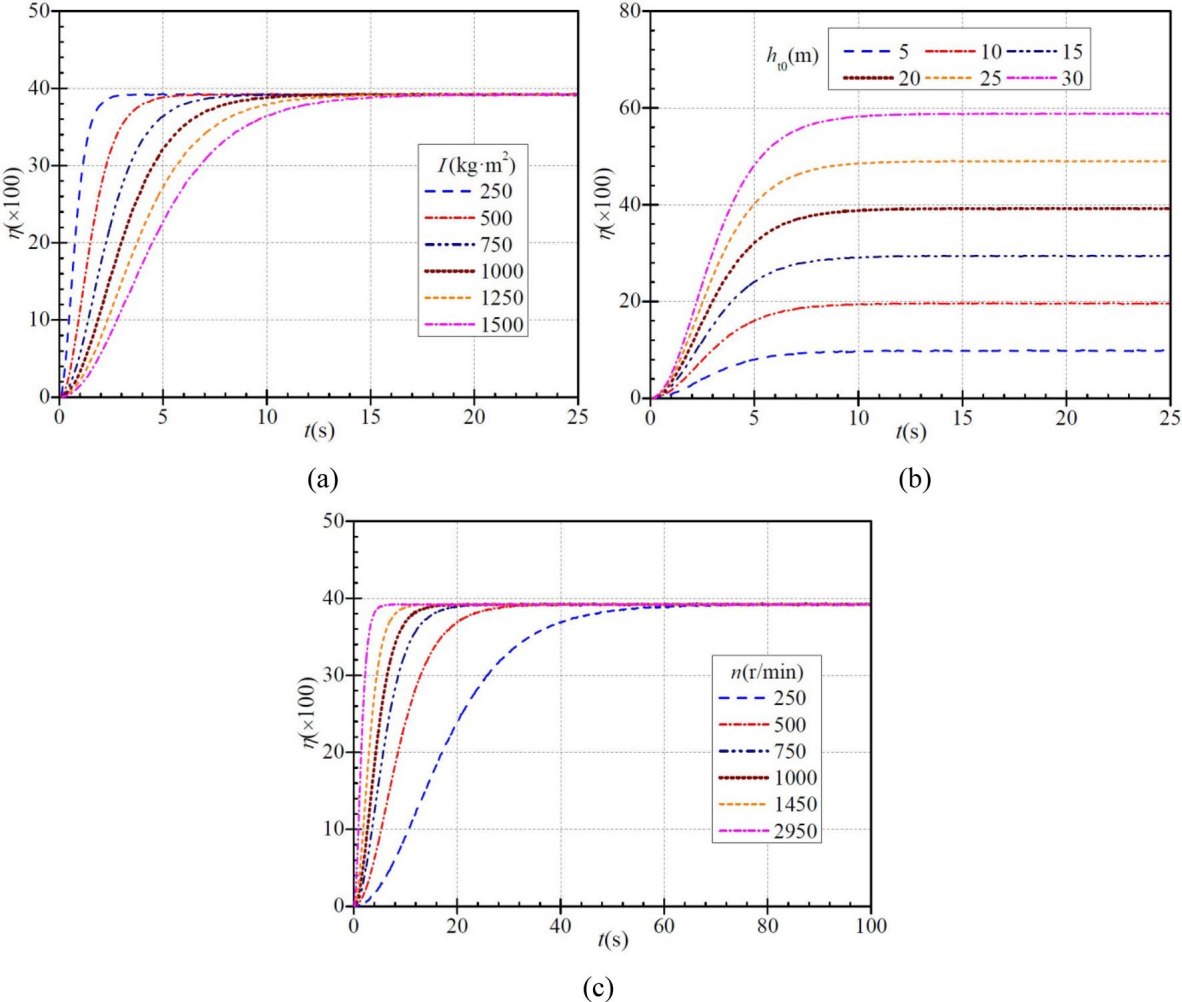
Instantaneous characteristics of conversion efficiency during PAT startup: (**a**) effect of moment of inertia; (**b**) effect of steady head; (**c**) effect of steady rotational speed.

In the PAT system, the PAT is used as an energy conversion element, and its energy conversion efficiency is very important for efficient energy utilization. The PAT conversion efficiency *η* is defined as

16 $$ \eta = \frac{P}{{P_{0} q}} = \frac{{\rho \text{g}h_{\text{t}} }}{{P_{0} }} $$

and Fig. 8 shows that *η* increases faster initially and then more slowly in each case.

Figure 8a shows that for each moment of inertia, the conversion efficiency increases gradually with time. For moments of inertia of 250 kg∙m^2^, 500 kg∙m^2^, 750 kg∙m^2^, 1000 kg∙m^2^, 1250 kg∙m^2^, and 1500 kg∙m^2^, the efficiency increases to a steady value of ca. 39.2% in a time of ca. 3.9 s, 6.7 s, 10.5 s, 13.3 s, 14.7 s, and 19.8 s, respectively. As can be seen, with increasing moment of inertia, the time required for the efficiency to reach the stable value increases, i.e., the rise becomes slower.

Figure 8b shows how the PAT stabilized head affects the efficiency. For each steady head, the efficiency increases gradually with time. When the stabilized heads are 5 m, 10 m, 15 m, 20 m, 25 m, and 30 m, the efficiency rises to the stabilized values of ca. 9.78%, 19.60%, 29.40%, 39.20%, 49.01%, and 58.80%, respectively, in a time of ca. 9.2 s, 10.9 s, 11.7 s, 13.3 s, 15.1 s, and 13.8 s, respectively. As can be seen, when the stabilized head is small (< 25 m), the time required to reach a steady efficiency becomes slightly longer.

Figure 8c shows that for each stabilized rotational speed, the efficiency tends to increase gradually with time. When the stabilized rotational speeds are 250 r/min, 500 r/min, 750 r/min, 1000 r/min, 1450 r/min, and 2950 r/min, the efficiency rises to the stabilized value of ca. 39.2% in a time of ca. 73.6 s, 37.6 s, 25.3 s, 19.0 s, 12.2 s, and 7.2 s, respectively. With increasing stabilized rotational speed, the efficiency increases more rapidly.

### Discussion

All the results presented above were obtained by changing one influencing factor while holding the others constant, from which some common and different features of transient PAT performance were identified. However, in practice, some factors such as flow rate and PAT head interact and work together, so in future work it will be very necessary to study how those interacting factors influence the transient PAT performance during startup. Moreover, the influence of pipe system and incoming flow on startup performance of PAT is also a work direction.

The proposed model is only able to predict the theoretical performance of PAT during startup or shutdown periods. In future works, the hydraulic loss model for PAT and pipe system during transient periods is also a working direction. Combining the proposed model and the hydraulic loss model, the actual performance would be obtained. Moreover, the experimental result is also necessary in future works so as to validate these model.

## Conclusions

In this study, the 1D unsteady Bernoulli equation for the piping system and the rotor angular momentum equation were used to derive a predictive model for the time-dependent performance of a PAT during startup, and this model can be applied to other transient processes. The conclusions of the study are as follows.

The instantaneous rotational speed, head, hydraulic power, and conversion efficiency of the PAT increase during startup, while the instantaneous flow rate and hydraulic head loss decrease, and both evolutionary trends have the characteristics of faster initially and then slower. During startup, the FAH increases initially and then decreases, but its value is negligibly small. With increasing rotor moment of inertia, the instantaneous rotational speed, head, hydraulic power, and conversion efficiency increase more slowly, but the flow rate and hydraulic head loss decrease more slowly. With increasing stabilized head of the PAT, the instantaneous flow rate and hydraulic head loss decrease more slowly. With increasing stabilized rotational speed of the PAT, the instantaneous rotational speed, head, hydraulic power, and conversion efficiency increase more rapidly, while the flow rate and hydraulic head loss decrease more rapidly.

## Data Availability

The data that support the findings of this study are available from the corresponding author upon reasonable request.
