# Correction: A theoretical model for predicting the startup performance of pumps as turbines

**DOI:** 10.1038/s41598-025-21687-y

**Published:** 2025-10-07

**Authors:** Yu-Liang Zhang, Yan-Juan Zhao, Zu-Chao Zhu

**Affiliations:** 1 College of Mechanical Engineering, Quzhou University, Quzhou, 324000 China; 2 College of Information Engineering, Quzhou College of Technology, Quzhou, 324000 China; 3 The Zhejiang Provincial Key Lab of Fluid Transmission Technology, Zhejiang Sci-Tech University, Hangzhou, 310018 China

Correction to: *Scientific Reports* 10.1038/s41598-024-57693-9, published online 23 March 2024

The original Article contained an error. In the section Theoretical Model, under the subheading ‘Unsteady Bernoulli equation for pump’, the sentence:

“Gao et al. established a mathematical model of the time-dependent hydraulic performance of a nuclear-reactor coolant pump during stopping period^14^.”

Now reads:

“Gao et al. established a mathematical model of the time-dependent hydraulic performance of a nuclear-reactor coolant pump during start-up period^14^.”

The original Article has been corrected.

